# The nocturnal acoustical intensity of the intensive care environment: an observational study

**DOI:** 10.1186/s40560-017-0237-9

**Published:** 2017-07-11

**Authors:** Lori J. Delaney, Marian J. Currie, Hsin-Chia Carol Huang, Violeta Lopez, Edward Litton, Frank Van Haren

**Affiliations:** 1 Faculty of Nursing, University of Canberra, Canberra, Australia; 2 College of Medicine, Biology and Environment, Australian National University, Canberra, Australia; 3 Respiratory and Sleep Medicine, Canberra Hospital, Canberra, Australia; 4 Alice Lee Centre for Nursing Studies, Yong Loo Lin School of Medicine, Singapore, Singapore; 5 St. John of God Hospital, Subiaco Perth Australia, Subiaco, Australia; 6 School of Medicine and Pharmacology, University of Western Australia, Perth, 6009 Australia; 7 Intensive Care Unit, Canberra Hospital, Canberra, Australia; 8 Faculty of Health: Discipline of Nursing, University of Canberra, Canberra, Act 2601 Australia

**Keywords:** Critical care, Disturbance, Intensive care, Noise, Sleep disturbance, Staff

## Abstract

**Background:**

The intensive care unit (ICU) environment exposes patients to noise levels that may result in substantial sleep disruption. There is a need to accurately describe the intensity pattern and source of noise in the ICU in order to develop effective sound abatement strategies. The objectives of this study were to determine nocturnal noise levels and their variability and the related sources of noise within an Australian tertiary ICU.

**Methods:**

An observational cross-sectional study was conducted in a 24-bed open-plan ICU. Sound levels were recorded overnight during three nights at 5-s epochs using Extech (SDL 600) sound monitors. Noise sources were concurrently logged by two research assistants.

**Results:**

The mean recorded ambient noise level in the ICU was 52.85 decibels (dB) (standard deviation (SD) 5.89), with a maximum noise recording at 98.3 dB (A). All recorded measurements exceeded the WHO recommendations. Noise variability per minute ranged from 9.9 to 44 dB (A), with peak noise levels >70 dB (A) occurring 10 times/hour (SD 11.4). Staff were identified as the most common source accounting for 35% of all noise. Mean noise levels in single-patient rooms compared with open-bed areas were 53.5 vs 53 dB (*p* = 0.37), respectively.

**Conclusion:**

Mean noise levels exceeded those recommended by the WHO resulting in an acoustical intensity of 193 times greater than the recommended and demonstrated a high degree of unpredictable variability, with the primary noise sources coming from staff conversations. The lack of protective effects of single rooms and the contributing effects that staffs have on noise levels are important factors when considering sound abatement strategies.

**Electronic supplementary material:**

The online version of this article (doi:10.1186/s40560-017-0237-9) contains supplementary material, which is available to authorized users.

## Background

Both sleep deprivation and fragmentation have been associated with a variety of adverse somatic, cognitive, and physiological effects. Patients are subjected to a cacophony of disruptive sounds in the intensive care unit (ICU) environment, and numerous studies have found an association between noise and the sleep disturbance experienced by patients [1–8]. Objective and subjective studies show that even small changes in noise levels can adversely impact sleep [1–4, 9]. For example, a 10 dB (A) (refer to list of abbreviations) increase in noise is perceived as a doubling of noise levels, while sound intensity is doubled for each 3 dB (A) increase [10]. Altered sleep architecture and circadian disturbances have been attributed to poorer patient outcomes and is potentially a precipitating cause for the onset of delirium [11–15].

There exists a number of official recommendations regarding noise levels within the hospital environment. The World Health Organization (WHO), the Environmental Protection Agency, and the International Noise Council all recommend that to reduce sleep disturbance, noise levels within hospital wards should not exceed 30 dB(A) at night [16]. However, none of these recommendations are specific to the ICU environment, but rather, they are generalised to the hospital environment [17].

Noises that may be disruptive to the ICU environment include conversations, monitor alarms, phone calls, doorbells, and door closures. Compared with lower acuity hospital areas, these are purported to be more frequent and intense in the ICU and result in elevated noise levels [17]. In addition to mean noise level, noise variability may also contribute to sleep disruption. Variations in noise levels may heighten the sympathetic nervous system response, increasing cortisol release, and increase sleep latency [6, 18]. Current research debates the impact of noise on the patients with polysomnography studies reporting that noise levels contributed to 15 to 20% of patient sleep disturbances [3, 4], in contrast to subjective studies which reported that noise is a significant stressor for patients [1, 5, 6, 11]. The effectiveness of interventions to reduce noise levels have been questioned with results revealing transient reductions in ambient noise levels, with limited reductions in the quantity of peak noise levels [1]. Emerging research suggests that the sound quality and the unpredictable variable changes in noise levels may be the major factor-associated adverse effects within the ICU environment which requires intervention [19, 20].

The primary aim of this study was to investigate the intensity and pattern of nocturnal noise levels such as variability and their sources within an Australian ICU compared with the recommendations stipulated by the World Health Organization (WHO). The secondary aim was to investigate whether single-patient rooms provided a significant reduction in ambient noise levels compared with open-bed areas.

## Methods

### Setting

**Fig. 1:**
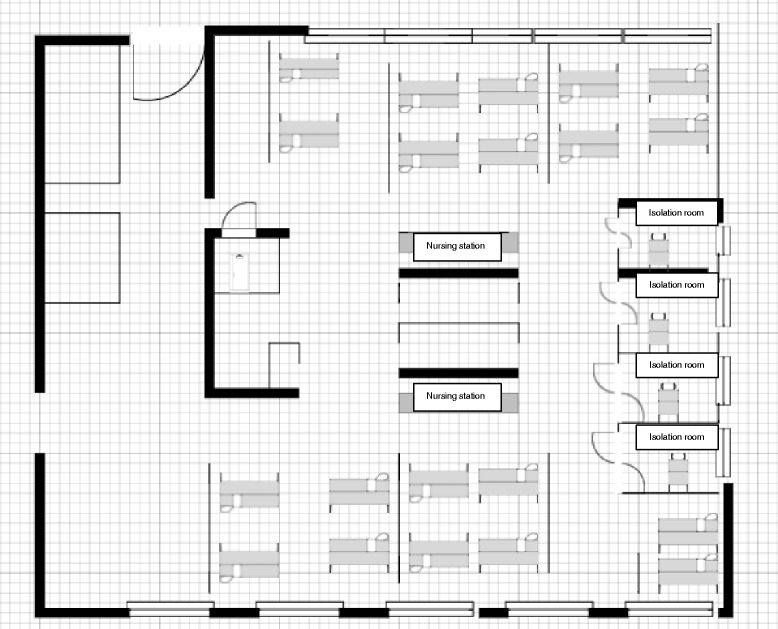
Schematic floorplan of the intensive care unit

This observational study was conducted in a 24-bed, open-plan designed, Australian tertiary referral ICU. The unit provides both ICU and high dependency care, including medical and surgical services, trauma and neurosurgical care and post-operative cardiothoracic care. The ICU design is characteristic of a second-generation ICU and includes two centralised open nursing stations, four isolation rooms, three two-bedded bays and four four-bedded bays, with all beds in shared bays separated by linen curtains and rooms separated by semi-partitioned walls (Fig. 1).

### Data collection procedures

#### Noise monitoring

Six sound monitors (Extech Model SDL 600) were positioned throughout the unit: at the nursing station and in the patients’ clinical environment. The placement of the sound meters was determined via cluster randomisation and involved only the occupied patient care spaces, with sites categorised as nursing stations, single-patient rooms, two-bedded and four-bedded bays. Sound monitors were mounted adjacent to the head of patients’ beds at a height of 155 cm. This position was chosen to reflect the experience of patients, minimise disruption to clinical care and reduce interference with other monitoring equipment. Noise levels were monitored for 9 h (2200–0700 h) over three weekday nights—two consecutive and one non-consecutive based on a predetermined hospital wide monitoring roster.

Sound levels were recorded in A-weighted decibels at 5-s epochs using an Extech Sound Level Monitor (Model SDL 600, frequency range 31.5 Hz–8 KHz, 30–180 dB (A)) which complied with the International Electro-technical Commission Standards. The A-weighted filter was used as it attenuates the curve that describes loudness frequency for the human ear. All logged data were saved onto a secured digital 2-GB memory card in a spreadsheet format for subsequent analysis.

### Logging of noise sources

Two research assistants completed the logging of noise sources in the ICU into predetermined categories: staff, alarms, doors, phones, wash basins, trolleys and pumps, including an ‘Other’ category to accommodate unexpected noise sources. To reflect the nocturnal activity in the clinical setting, the research assistants logged noise sources at four different time points: 2300 to 2400, 0200 to 0300, 0400 to 0500 and 0600 to 0700 h.

### Data analysis

Data derived from the sound monitors (Extech Model SDL 600) were downloaded and exported to Microsoft Excel (2010). Descriptive statistical analysis (means and standard deviations) was undertaken using IBM SPSS software (version 20) to describe the noise levels recorded. The variability of noise levels within the ICU were calculated by determining the difference between the mean and maximum noise levels within 5-min intervals over the 9 h recording period. The noise sources logged by the research assistants for each hour were collated and reported via descriptive statistics. An analysis of variance (ANOVA) was performed to identify differences between noise levels recorded over each of the three nights and to determine if single-patient rooms were significantly quieter than shared patient spaces.

The perceived loudness of noise is a logarithmic measure and subsequently does not exhibit a linear relationship to changes in noise levels. In order to quantify the impact of the recorded noise levels, psychoacoustical analysis was undertaken in order to describe the increases in volume and the acoustical intensity that these produce (Additional file 1: Table S1) [18]. Identification of these measures provides details of sound levels produced in the environment, whilst acoustical intensity reports a linear relationship of how many times greater the noise levels are compared to the recommended levels (30 dB A).

### Ethics

The Australian Capital Territory Health Human Research Ethics Committee (ETHLR.12.253) approved the study; the need for informed consent was waived. The study was conducted in accordance with the principles outlined in the Declaration of Helsinki.

## Results

**Fig. 2:**
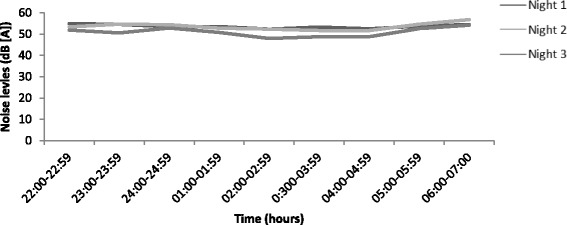
A comparison of mean noise levels over the three nights of monitoring

A total of 18 clinical spaces, which included 16 patient care spaces and two nursing stations between June and September 2013 were monitored for nocturnal noise levels. Each bed space was occupied for the entire monitoring period. The ambient noise levels recorded over the three nights were similar with a variance of 2.86 dB (A) (night 1 *L*_A mean_ = 53.71 ± 4.69 dB (A), night 2 *L*_A mean_ = 53.15 ± 6.21 dB (A) and night 3 *L*_A mean_ = 50.85 ± 6.18 dB (A)) (Fig. 2). The overall ambient nocturnal noise level for the monitoring period in the ICU was 52.85 dB (A) (SD 5.89) and exceeded the WHO recommendations by 22.85 dB (A), which produces an increase in both noise volume (4.8 times greater) and acoustical intensity (192.8 times greater) than recommended.

The peak noise levels ranged from 85.5 to 98.3 dB (A). Noise levels within single-room and open-bed areas were similar to noise levels in two-bedded bays (*p* = 0.37) and four-bedded bays (*p* = 0.06) (Table 1). The primary sources of environmental noise were staff conversations and monitor alarms, which accounted for 35.4 and 34.1% of noises per hour respectively (Table 2).

**Table 1:** Nocturnal noise levels within the ICU

Location	Mean (dB (A))(SD)	Min (dB (A))	Peak (dB (A))
Intensive care unit (overall)	52.9 (5.9)	40.2	98.3
Nursing station	54.8 (5.8)	42	98.3
Isolation rooms	53.5 (4.1)	47.7	96.3
Two-bedded bay	53 (6.8)	41.6	86.1
Four-bedded bay	52 (6.1)	40.2	85.5

**Table 2:** Sources of nocturnal noise in occurrences per hour in the ICU

Source	Mean occurrences per hour mean (SD)	Proportion of total occurrences per hour (%)
Staff	84.38 (32.77)	35.5
Alarms	81.06 (43.95)	34.1
Other	35.81 (19.96)	15.1
Doors	12.88 (12.37)	5.4
Pumps	7.19 (12.55)	3.0
Equipment	6.06 (4.64)	2.6
Trolleys	5.94 (7.19)	2.5
Wash basins	4.13 (2.55)	1.7

**Fig. 3:**
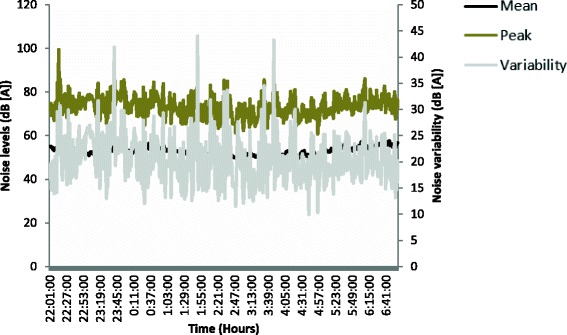
Noise patterns in the intensive care unit. Mean noise (dB (A)) levels for each 5-min interval are presented for the 9 h of recording. Comparatively, the maximum noise levels recorded for each of the intervals are depicted, identifying peak noise levels (dB (A)) that patients are exposed to during the nocturnal period. Variability in noise levels (dB (A)), as determined by the difference between the mean noise level and the maximum noise level for each 5-min interval is presented and reflects the rapidly changing noise levels present in an ICU environment

The variability in ambient noise levels in the ICU is shown in Fig. 3, with frequent undulations in noise levels ranging from 9.9 to 44 dB (A) above ambient noise levels. Peak noise levels >70 dB (A) were found to occur 10 times/hour (SD 11.4).

## Discussion

### Noise

We found that nocturnal noise exceeded the international recommendations throughout the entire monitoring period. The noise levels were of similar intensity to heavy traffic. Our results are consistent with other studies reporting noise levels in an ICU [1, 4, 6, 21–24]. The elevated ambient noise levels and the high degree of variability, in combination with the frequency of peak noises identified, are likely to contribute to sleep disruption [1–3, 21–23]. At the reported noise levels, the ICU environment is perceived to be 4.8 times louder, producing an acoustical intensity 193 times greater than the WHO recommendations. Our ICU can be classified as moderately noisy (50−60 dB (A) to very noisy (60−70 dB (A)) according to the hospital noise levels described by Pereira et al. [24] and may have adverse implications for patients in the ICU.

Sleep disturbances associated with waking, arousal and sleep-to-wake transitions are purported to occur with sound intensities ranging between 50 and 60 dB [25, 26], with spikes in noise levels identified by Gabor et al. [4] as a contributing factor to sleep disruption. Stanchina and colleagues demonstrated that noise variability between ambient noise levels and peak noises determined the number of arousals for individuals exposed to ICU noise [19]. This indicates that noise disturbance is not only attributed to peak noises, but is also associated with frequency and unpredictability [27]. Subsequently, the capacity for patients to acclimatise to noise levels is unlikely to occur when the noise levels generated demonstrate frequent undulations and elevations in noise levels. The variability in noise levels within the ICU is likely to preclude patients’ ability to acclimatise to noise within the environment and adversely impact on sleep continuity, contributing to additional physical and psychological stress. This is supported by patient reports, whereby unfamiliar and loud noises where a major causative factor in preventing their ability to sleep whilst in the ICU [28].

The resultant unpredictability of the environment has been associated with a heightened stress response secondary to autonomic stimulation and enhanced sympathetic activity, which adversely affects sleep latency. As a result, increased cortisol release inhibits melatonin secretion and thereby disrupts circadian rhythm regulation [29]. Exposure to noise sources greater than 50 dB (A) have been shown to produce cardiovascular changes such as increased heart rate variability, [30–32] along with electroencephalographic changes suggestive of arousal and lightening of sleep [33]. The association between heart rate and acoustics was reported by Hagerman et al. who identified an increase in pulse rate occurring with higher acoustical exposure, resulting in an increased incidence of readmission in a coronary care unit [34]. The variable and unpredictable changes in noise levels within the ICU are likely to impede on sleep quality and continuity, which may have further implications on cognitive function and the development of delirium.

Further, clinicians working within the environment are also susceptible to noise-induced stress, which can lead to exhaustion and irritability [35]. The emerging research on ‘alarm fatigue’ suggests that 72 to 99% of clinical alarms are false alarms. This contributes to staff desensitisation to alarms and has even been associated with patient deaths [36]. Further, evidence suggests that noise can impinge on concentration and clinical decision-making [36].

The use of single-patient bed spaces as a means to reduce patients’ exposure to elevated and disruptive noise levels is not supported by the findings of this study, with four-bedded bays having the lowest ambient noise levels. Previous findings reported by Tegnestedt et al. further support this finding, whereby single-patient rooms were not found to have lower noise levels than shared patient spaces [37]. The reported noise levels in the single-patient rooms in this study may be reflective of the acuity of patients cared for in these rooms. Higher acuity patients require greater clinical interventions resulting in more noise being generated, whilst four-bedded bays may be more likely to be co-habited by less acute patients. In addition, behavioural modifications, such as regulatory processes that staff engage in, in shared care spaces may contribute to a more conscientious approach to the noise generated in order to reduce its burden and impact on multiple individuals. Further, the clinical design of the single-patient rooms may also be a contributing factor, whereby alarm volumes may need to be increased in order to assure staff’s ability to hear them when outside of the room or in the anteroom. In addition, the lack of noise-absorbing features may be a critical consideration that results in greater noise reverberation.

### Sources of noise

The frequent ascension of noise levels in this study was observed to be associated with frequent monitor alarms, to which staff did not always respond. The impact of this on environmental noise was compounded by the ability for staff to communicate intelligibly to safeguard clinical decision-making and interventions, whereby speech needs to be 15 dB greater than the ambient noise to ensure clarity of communication. This in turn contributes to a further escalation in noise levels, known as the Lombard effect [38]. Noises produced at these levels are unlikely to provide patients with a nocturnal environment to support sleep and is problematic for clinical staff to ensure that communication is intelligible to mitigate the risk of error. This may account for the high rate of noise attributed to staff behaviour and suggests that moderating behaviour and alarm settings may have a beneficial effect on reducing nocturnal noise levels. The reported noise levels and the identified sources of noise in this study may adversely affect both patient and clinicians, and thus, there is a need for strategies to be devised and implemented to reduce the disruptions and stressors imposed by constant noise exposure.

### Possible interventions

The possibility of curbing noise levels in the ICU environment by employing behaviour modification approaches to ameliorate the noise generated by staff and alarms has been previously identified. Such initiatives have included modifying alarms (tailoring parameters to patients and adjusting the volume at night) [1, 39] staff education regarding the physiological aspects of sleep and its role in recovery [39, 40] and behaviour-regulating interventions such as the use of ‘yacker trackers’ to identify noise increases [41]. While many of these studies report an initial successful reduction in subjective noise levels, few have demonstrated measurable or sustained reductions [1, 38, 41–44]. These studies suggest that strategies need to consider a range of approaches including sound elimination such as providing patients with earplugs and re-designing the environment rather than focusing solely on behaviour modification [29, 41, 44].

Research regarding environmental design indicates that it has the capacity to influence psychology, physiology and social behaviours, which may contribute to a reduction in noise [45].A landmark report published by Ulrich and colleagues [29] identified 600 studies which linked the clinical environment to positive or negative outcomes for patients and staff in four main areas: patient safety; staff stress and fatigue; increased effectiveness in care delivery; and improved overall healthcare quality. Specifically, these authors found that design features such as single-patient rooms, noise-absorbing materials and exposure to natural light to be useful in promoting appropriate circadian rhythms and reducing noise to facilitate sleep. Other authors have found that these design features were associated with a reduction in noise and improved sleep [4, 46–48], a reduction in medication errors [49, 50], improved communication [51] and reduced length of hospital stay [51–53]. However, despite the reported benefits, fiscal restraints imposed on healthcare organisations have resulted in selective implementation of recommendations [41].

### Limitations

The findings of this study cannot be generalised to all ICU environments, as the study was conducted in a single Australian ICU, which may be unique in design and location. The noise levels recorded were recorded at night and are likely to be lower than the noise levels during the day. Further, the study did not undertake direct patient assessment regarding the impact of noise on ICU patients, and as a result, the impact of noise can only be postulated based on the current findings and previous research. The presence of the observer and environmental-monitoring equipment in the clinical environment could have contributed to alterations in behaviour in order to conform to the presumed research objectives. If anything, this is likely to underestimate true noise levels. Despite these limitations, the study included the measurement of noise from multiple locations within the ICU, across three separate nights, and the identification of noise variability which may be an independent risk factor for noise-induced harm. The concurrent logging of noise sources during the monitoring period permitted the identification of primary noise sources rather than speculating on the primary contributing factors.

## Conclusion

The occurrence of high mean noise levels in combination with the variability in noise levels and the frequency of peak noises may contribute to sleep disruption. Mean nocturnal noises in the ICU significantly exceeded those recommended by the WHO and demonstrated significant variability which is likely to result in substantial sleep disruption. The primary sources of noise were identified as staff conversations and monitor alarms. Single rooms had similar noise levels to those of two- and four-bedded bays. Further research into strategies to reduce noise, the physiological responses (e.g. heart rate, cortisol levels) and evaluation of interventions is required to enhance the therapeutic environment and understand their implications on patients. Providing patients with both relief from persistent exposure to noise and diurnal variation in noise levels have the potential to aid in their physiological and psychological recovery.

## Additional file

Additional file 1: Table S1. Sound level change and loudness ratio. (DOCX 15 kb)

## Abbreviations

ANOVA: Analysis of variance
dB (A): Decibels in A-weighted scale
ICU: Intensive care unit
*L*_A mean_: Mean ambient noise
SD: Standard Deviation
SPSS: Statistical Package for Social Science
WHO: World Health Organization
