# Supplementary material for: The nocturnal acoustical intensity of the intensive care environment: an observational study
Source: J Intensive Care. 2017 Jul 11;5:41. doi: 10.1186/s40560-017-0237-9 (PMC5504755; doi:10.1186/s40560-017-0237-9)
Supplement: Additional file 1: Table S1. — Sound level change and loudness ratio. (DOCX 15 kb) [file 40560_2017_237_MOESM1_ESM.docx]

**Additional file 1: Table S1. Sound level change and loudness ratio**

|  | Psychoacoustics | Volume | Acoustical Intensity |
| --- | --- | --- | --- |
| Equation | ∆ *L*=10∙log_2_χ = 32.22∙log_(χ)_ | χ = 10$\frac{\boldsymbol{\Delta L}}{\boldsymbol{32.22}}$ = 2$\frac{\boldsymbol{\Delta L}}{\boldsymbol{10}}$ | Z= 10$\frac{\boldsymbol{\Delta L}}{\boldsymbol{10}}$ |
| Outcome | Level change ∆ *L*_loud_ = 22.85 | 4.87 times louder | 192.75 times greater |
